# Substrate-driven optimization of microfluidic aluminum–air fuel cells: a comparative study of glass fiber vs. cellulose paper

**DOI:** 10.1038/s41598-026-50431-3

**Published:** 2026-05-09

**Authors:** Purshottam J. Assudani, R. Lavanya, Srinivas Samala, Ch. Rajendra Prasad, M. Karthik, Prakash Rewatkar, Manish Bhaiyya, Madhusudan B. Kulkarni

**Affiliations:** 1 School of Computer Science and Engineering, Ramdeobaba University, Nagpur, Maharashtra India; 2 Department of Computing Technologies, SRM Institute of Science and Technology, Kattankulathur, Tamil Nadu India; 3 Department of ECE, SR University, Warangal, Telangana 506371 India; 4 Department of Electrical and Electronics Engineering, Kongu Engineering College, Erode, Perundurai, Tamil Nadu India; 5 Department of Mechanical Engineering, Israel Institute of Technology, 3200003 Haifa, Israel; 6 Department of Electronics and Telecommunication Engineering, Shri Sant Gajanan Maharaj College of Engineering, Shegaon, MH 444203 India; 7 Manipal Institute of Technology, Manipal Academy of Higher Education, Manipal, India

**Keywords:** Microfluidic Al–air fuel cell, Paper-based fuel cells, Glass fiber paper, Electrolyte transport, Power density optimization, Energy science and technology, Engineering, Materials science

## Abstract

Microfluidic aluminium–air fuel cells (µA–aFCs) offer attractive low-cost, compact, and environmentally benign power sources for portable and off-grid electronics. Their performance is strongly governed by electrolyte transport within the paper-based microfluidic channel. Here we demonstrate that the performance of µAl–aFCs can be significantly improved by replacing conventional cellulose (Whatman #1) electrolyte substrates with glass fiber (GF) paper. Owing to its more open fibrous network, accelerated capillary flow, and improved chemical stability under alkaline conditions, GF paper enables more efficient electrolyte replenishment to the cathode and facilitates ionic transport within the device. Using KOH and NaOH electrolytes ranging from 0.5 to 5 M, electrochemical polarization, impedance, and discharge characteristics were evaluated as a function of substrate type and electrolyte concentration. The optimal configuration, consisting of GF paper and 3.5 M KOH, produced a peak power density of 72.89 ± 1.66 mW/cm² and a maximum current density of 143.76 ± 1.14 mA/cm², nearly double that obtained with Whatman #1 substrates. Impedance analysis indicates reduced ohmic and diffusion-related losses at intermediate electrolyte concentrations, while galvanostatic discharge tests demonstrate stable operation under constant load. These findings highlight substrate engineering using GF paper as a simple materials-based strategy for enhancing the performance of microfluidic Al–air fuel cells.

**Supplementary Information:**

The online version contains supplementary material available at 10.1038/s41598-026-50431-3.

## Introduction

Novel microfluidics implementations into Al–air fuel cell technology have also been reported microfluidic aluminium-air fuel cells (µA–aFCs)^1–3^. Such miniaturisation and system simplification is achieved by permitting electrolytes to be delivered passively to the electrochemical interface by means of capillary action within porous materials, employing the wicking properties of paper to deliver electrolyte without the need for pumps, valves, or active controls. This not only reduces system complexity and cost but also enhances the portability and sustainability of the fuel cell architecture^4^. This design is usually achieved by inserting a porous paper layer in between an aluminium anode and an air-breathing cathode with an aqueous alkaline electrolyte, such as potassium or sodium hydroxide (KOH or NaOH), acting as the ionic conductor^5,6^. Recent studies have also explored microscale transport phenomena and electrokinetic effects in microfluidic systems, highlighting the important role of interfacial transport processes in governing fluid behaviour and species distribution at small length scales^7–10^.

In alkaline media, the electrochemical reactions governing the operation of the Al–air fuel cell are as follows:

Anode (Al oxidation):

$$Al + 4OH^{ - } \to Al(OH)_{4}^{ - } + 3e^{ - }$$

 Cathode (Oxygen reduction reaction, ORR):

$$ O_{2} + 2H_{2} O + 4e^{ - } \to 4OH^{ - } $$

Overall cell reaction:

$$ 4Al + 3O_{2} ~ + {\text{ }}6H_{2} O \to 4Al(OH)_{3} $$

In both KOH and NaOH electrolytes, hydroxyl (OH⁻) ions act as the active ionic species, participating directly in the anodic oxidation of aluminium and regeneration at the cathode during oxygen reduction.

While the concept of paper-based µA–aFCs has great potential for general use, there are currently many limitations due to the low level of power generation and inability to provide consistent long-term usage^11,12^. One primary reason for these limitations is that the electrolyte transport mechanism controls how ions move through an electrode and electrolyte interface; this in turn affects how the electrochemical reaction occurs over time^13^. In addition, the performance of µA–aFCs is also dependent upon the physicochemical properties of the substrate material, including its porosity (degree of openness), pore size, thickness, and its inherent capillarity mechanism. Conventional materials used in these systems comprised predominantly of cellulose-based substrates, such as Whatman #1 filter paper, have a proven record for being inexpensive, biodegradable, and having a moderate amount of capillary action^14,15^. Nevertheless, the performance of these materials is drastically reduced when working with high concentrations of electrolyte because of their ability to wick moisture very slowly, clog pores when saturated, and lose structural integrity when exposed to alkaline conditions for extended periods^16,17^.

Recent publish studies on paper-based and microfluidic Al–air systems have mainly focused on improving electrode materials, catalysts, and electrolyte type, yet these studies still depend on cellulose substrates that impose inherent transport constraints. Generally, cellulose-based µA–aFCs will normally exhibit low power densities and they exhibit a significant drop in efficiency as the concentration of the electrolyte is increased^18,19^. The decrease in efficiency is believed to be due to the slow diffusion of hydroxyl ions through the cellulose material and the low amount of fresh electrolyte being delivered to the cathode under these conditions. Even though electrolyte transport is an important factor in determining ohmic resistance (Rs), charge-transfer resistance (Rct), and losses due to diffusion, there has been very little focus on the substrate, which has rarely been developed as performance booster^20,21^.

To overcome these key limitations, this work investigates the use of glass fiber (GF) paper as an advanced electrolyte transport medium in µA–aFCs. The GF paper, made from interwoven micro glass fiber, offers several advantages over traditional cellulose substrates such as greater chemical resistance, superior thermal stability, higher more open fibrous network, and markedly enhanced capillary-driven electrolyte transport^22,23^. Beyond its structural advantages, GF paper demonstrates superior wetting and absorption characteristics, which are vital for sustaining a steady and uniform electrolyte flow to the cathode during prolonged operation. Its mechanical strength under high-concentration alkaline environments further supports consistent and efficient ion transport over time, effectively mitigating issues such as pore blockage, capillary collapse, and structural deterioration that frequently occur in conventional substrates like Whatman #1 filter paper^24,25^. However, despite the potential benefits of GF substrates, no prior study has thoroughly compared glass fiber and cellulose papers in the context of µA–aFCs, or examined how substrate selection dictates electrolyte transport, and components of internal resistance providing a notable gap in the knowledge base on the topic^13,26^. Such a gap is highly significant, since substrate driven capillarity plays an important role in hydroxyl-ion mobility, and thickness of diffusion-layer at the cathode, as well as the kinetics of aluminium oxidation at the anode.

To the best of our knowledge, this work for the first times combines substrate engineering with electrolyte optimization to enhance the µA–aFC performance. In particular, (i) probe the transport and electrochemical behaviour of GF paper versus conventional Whatman #1 paper, (ii) investigate the effect of KOH and NaOH electrolytes in a wide range of concentrations (0.5–5 M), and (iii) assess the most effective working conditions for optimizing power density and current output. Through a combination of polarization, electrochemical impedance spectroscopy (EIS), discharge analysis, and microscopic characterization, it is evident that GF paper not only promotes capillary-driven electrolyte, transport, but also minimizes transport losses and enhances cell efficiency. These results position GF paper as an attractive substrate for high performance, reliable, and microfludically scalable microfluidic Al–air fuel cells. By treating substrate selection as a materials parameter rather than a passive component this work opens new routes towards next-generation miniaturised, portable, and off-grid energy for IoT modules, disposable diagnostics, environmental sensors, and emergency electronics.

## Methods section

### Materials and chemicals

The Platinum/Carbon catalyst (2 mg/cm^2^) was purchased from Fuel Cell Store Systems, USA. The Glass microfiber filter paper (GF/A, 260 μm thickness, 1.6 μm pore size) and Whatman # 1 (180 μm thickness, 11 μm pore size) filter paper was procured from Whatman, Israel. Both substrates were supplied as circular filter paper discs with a diameter of 90 mm. The typical laboratory cost of a Whatman No.1 cellulose filter paper (90 mm diameter) is approximately 0.18 USD per piece, whereas the glass fiber filter paper (GF/A, 90 mm diameter) costs approximately 2.15 USD per piece. The food-grade aluminium foil (25 μm) was procured from the grocery store. The electrolyte potassium hydroxide (KOH) and sodium hydroxide (NaOH) were bought from Biolab Chemicals Ltd. Israel. The solutions were prepared in the Milli-Q (18.2 MΩ cm) water and materials in the experiment were used directly without further modification and purification.

### Fabrication and realization of Al-air fuel cell

**Fig. 1:**
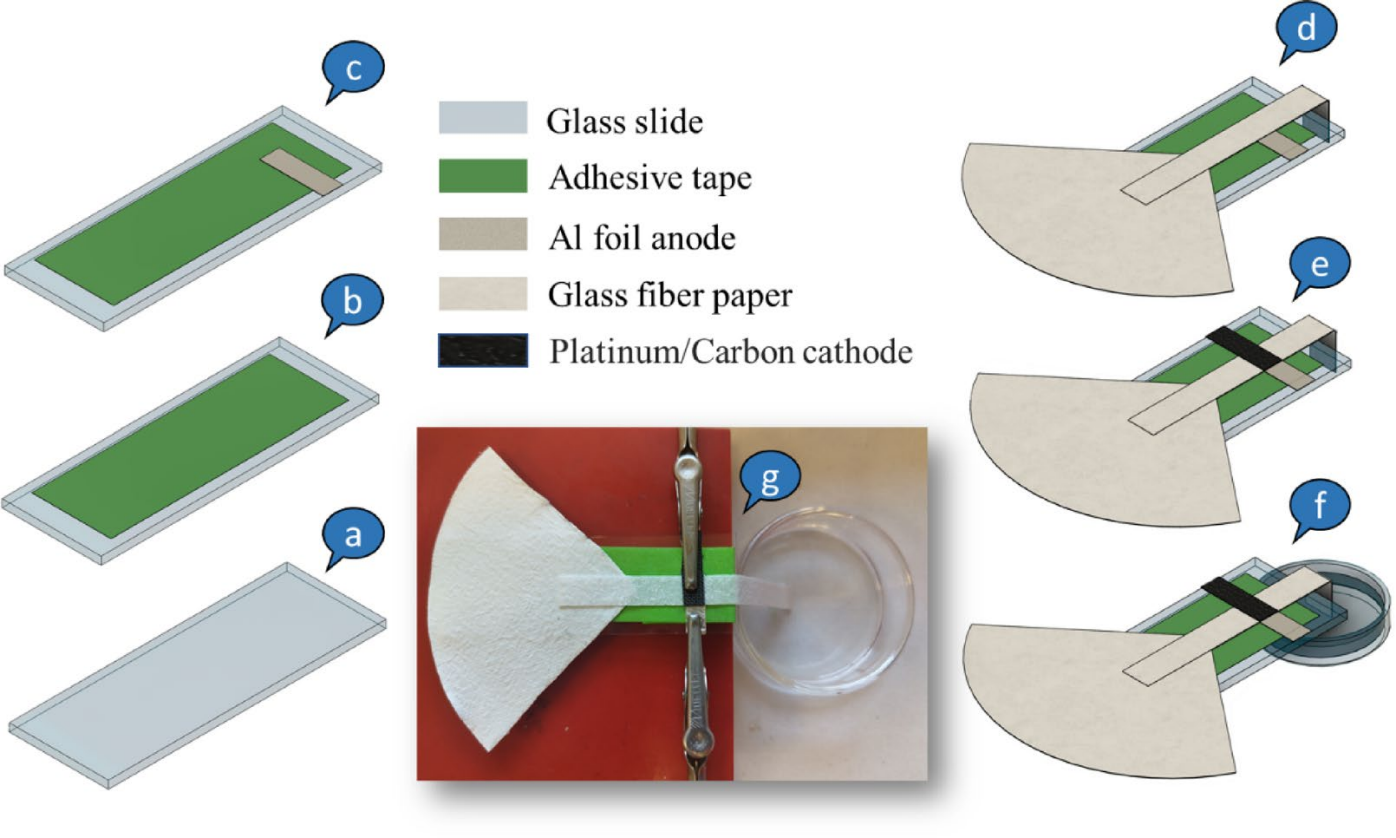
Presents a stepwise schematic of the GF paper-based µA–aFC, showing: (**a**) the microscopic glass slide support, (**b**) the adhesive tape layer, (**c**) the aluminium-foil anode, (**d**) the paper-based microchannel and absorbent pad, (**e**) the platinum/carbon catalyst cathode, (**f**) the assembled µA–aFC loaded with aqueous electrolyte, and (**g**) a real-time photograph of the operating GF paper-based device.

The device consists of an Al foil (anode), filter paper as an aqueous electrolyte substrate, and, Platinum/Carbon catalyst (cathode) which was assembled in the described manner. The inexpensive Al foil (98.2% purity, food grade) was directly used with an active area of 0.5 cm^2^. Porous carbon cloth (2 mg/cm^2^) was used for the cathode. As for electrolyte substrate Glass microfiber filter paper (GF) was used and engraved via laser system (VLS 3.6 W, Universal laser system, USA) in 7 cm in length × 0.7 cm width. The active area (0.5 cm²) was defined by the overlap between the aluminium anode, the laser-patterned paper electrolyte channel, and the Pt/C cathode during assembly. The paper substrate was laser patterned, while the aluminium anode and carbon cloth cathode were manually cut to the marked dimensions to match the channel region, ensuring consistent alignment and a reproducible active area. The schematic diagram of stepwise GF paper based µA-aFC fabrication and realization shown schematically in Fig. 1.

### Electrochemical characterization

Biologic Potentiostat/Galvanostat (VSP, France) was employed for conducting all the electrochemical, polarization and discharge studies. The AC impedance (electrochemical impedance spectroscopy, EIS) experiments were performed in a two-electrode configuration and at open-circuit conditions (zero DC current) within the frequency range of 100 kHz to 300 mHz. The polarization curve was obtained via I–V characterization by sweeping the cell potential from the open-circuit potential (OCP) to 0.1 V at a scan rate of 50 mV/s. The lower potential limit of 0.1 V was selected to avoid cell instability or collapse that may occur at near-zero voltage conditions, while also minimizing electrolyte depletion and maintaining stable capillary-driven electrolyte supply during measurement. Likewise, the stability analysis was pursued by discharging the cell at a constant current density of 1, 10 and 20 mA/cm^2^. All electrochemical measurements were performed under ambient laboratory conditions (approximately 25 ± 2 °C). The devices were operated without hermetic sealing; however, the electrolyte retained within the porous paper substrate provided sufficient liquid retention to minimize noticeable evaporation during the relatively short polarization and discharge measurements.

## Results and discussion

### Microscopic electrode surface characterization

**Fig. 2:**
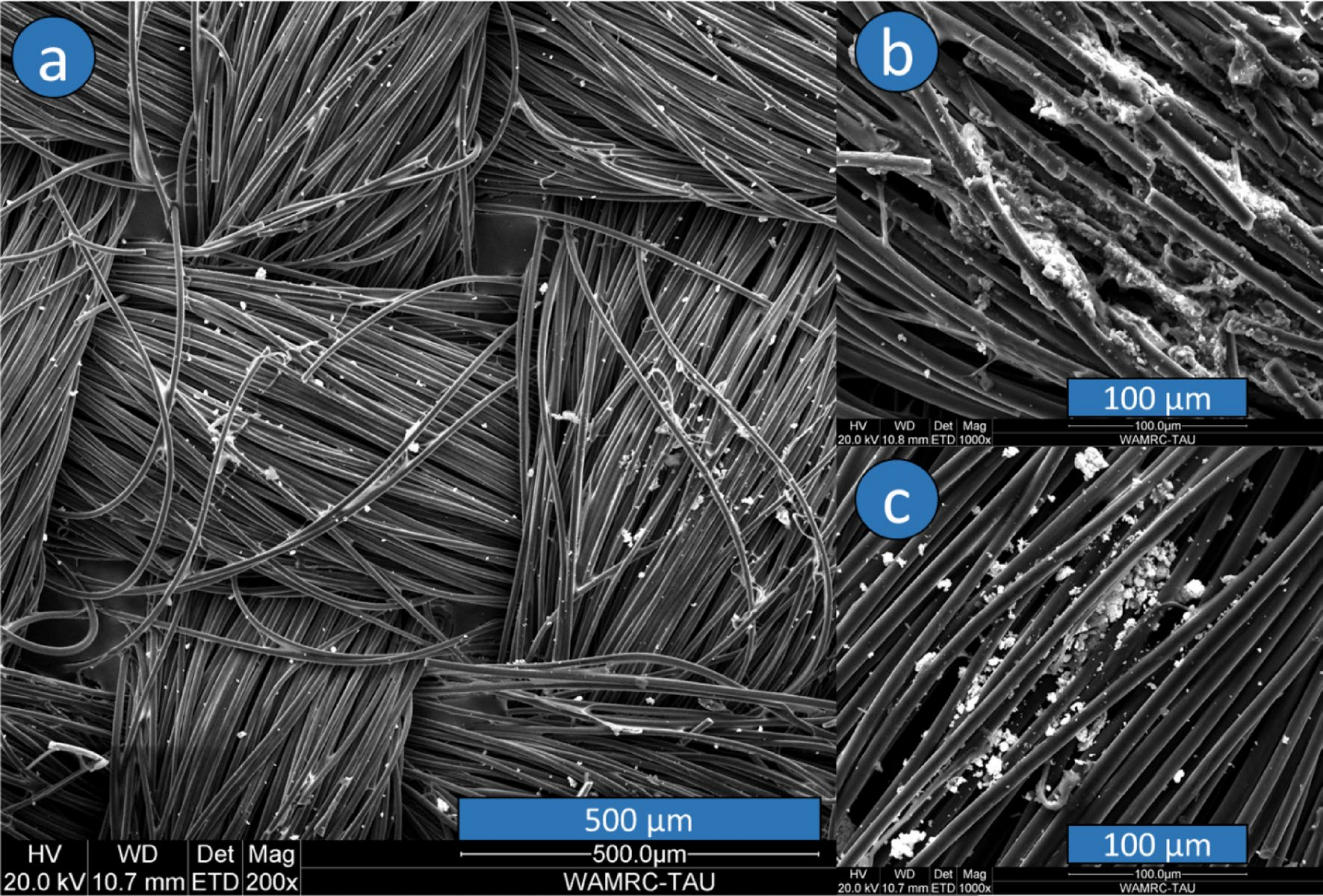
SEM images of the Pt/C carbon fabric cathode: (**a**) bare electrode before electrochemical testing showing the woven carbon fiber structure; (**b**) electrode surface after operation in KOH electrolyte, where surface deposits are observed along the carbon fiber; and (**c**) electrode surface after operation in NaOH electrolyte, also showing the formation of precipitate deposits associated with reaction products formed during Al–air fuel cell operation.

Scanning electron microscopy (SEM) was employed to examine the surface morphology of the electrode before and after the electrochemical experiments. Such analyses provide insight into electrochemical reaction processes through the observation of structural changes and surface deposits formed during operation. After the polarization experiments, the cathode was carefully removed from the fuel cell, gently rinsed with deionized water to remove residual electrolyte, and dried under ambient conditions prior to SEM characterization. Figure 2a shows the carbon fabric composed of woven micro-yarn bundles prior to the experiment. After electrochemical testing, as shown in Fig. 2b and c, the original fibrous micro-yarn structure of the carbon fabric remains largely intact, indicating that the electrode maintains its structural integrity despite undergoing multiple electrochemical reactions. However, noticeable precipitate deposits are observed along the carbon fiber.

To investigate the composition of these surface deposits, energy-dispersive X-ray spectroscopy (EDS) analysis was performed on the bare and post-tested cathodes, and the results are summarized in Table 1. The bare Pt/C electrode primarily exhibits signals corresponding to C and Pt, associated with the carbon cloth substrate and the Pt/C catalyst layer, respectively. A small amount of F is also detected, which is attributed to the fluorinated binder commonly used in carbon-based gas diffusion electrodes. After cell operation, the EDS spectra reveal the presence of Al and O, together with electrolyte-specific cations, namely Na in NaOH electrolyte and K in KOH electrolyte, in addition to C and Pt from the electrode material. The increased oxygen content and the appearance of aluminium indicate the formation of aluminium-containing alkaline reaction products on the cathode surface during fuel cell operation.

The apparent reduction in the detected Pt content after operation is attributed to partial coverage of the Pt/C catalyst layer by deposited reaction products. Since SEM–EDS provides relative elemental composition from the beam-interaction volume, the appearance of additional Al, O-, Na, or K-containing species on the cathode surface decreases the relative Pt fraction detected in the post-test spectra. The F signal observed for the bare cathode is consistent with the presence of a fluorinated binder such as PTFE, whereas its absence after operation is plausibly due to localized masking of the underlying binder-rich regions by the deposited products. Because EDS provides elemental rather than definitive phase information, the deposits are described here as aluminium-containing alkaline or aluminate-related surface products rather than being conclusively assigned to a single phase^27,28^.

The hydrated aluminium-containing precipitates observed in Fig. 2b and c are consistent with the electrochemical oxidation of aluminium in alkaline electrolytes, which leads to the formation of aluminate-related products in the presence of KOH or NaOH electrolytes. These reactions proceed until the aluminium fuel is consumed, resulting in progressive accumulation of reaction products on the electrode surface, as reported in our previous studies^25,^.

**Table 1:** EDS elemental composition of the Pt/C cathode before and after operation in KOH and NaOH electrolytes.

Elements (K)	Bare Pt/C cathode (wt%)	Presence of KOH (wt%)	Presence of NaOH (wt%)
C	75.93	68.09	69.75
O	9.47	21.44	20.25
Pt	8.37	3.45	2.74
Na	-	-	4.98
K	-	4	-
Al	-	3.02	2.29
F	3.23	-	-

### Substrate selection and analysis

**Fig. 3:**
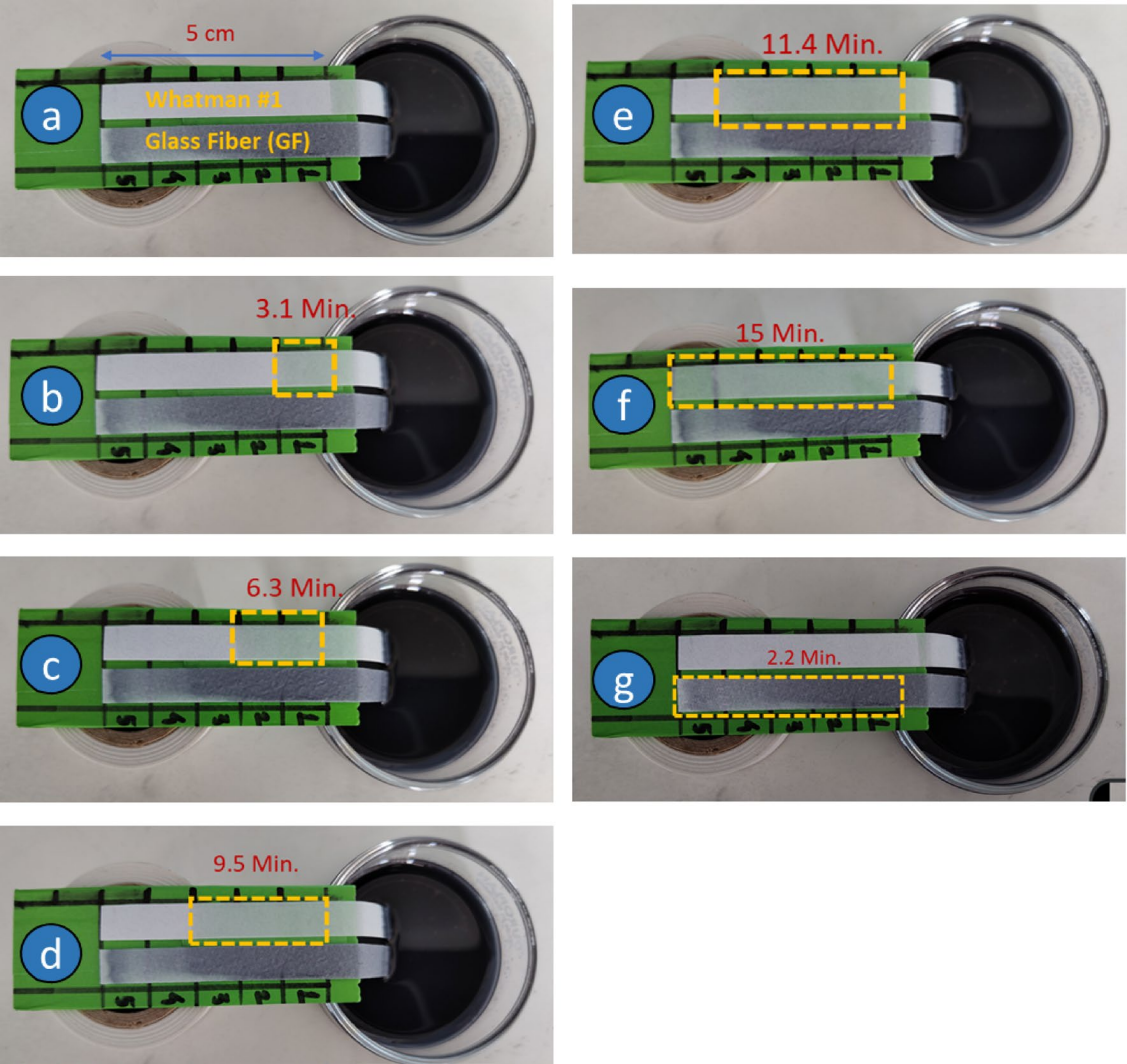
Wicking behaviour of different paper substrates used in Al–air fuel cells. (**a**) Photograph of a 5 cm long paper strip prepared from Whatman #1 and GF paper. (**b**–**g**) Time-lapse images comparing the fluid transport behaviour in the two substrates. Whatman #1 requires approximately 15 min to reach the 5 cm distance (**f**), whereas GF achieves the same distance in only 2.2 min (**g**), demonstrating significantly improved capillary transport.

The transport of aqueous electrolytes primarily occurs through the paper substrate itself. The porous and permeable nature of paper allows for capillary action and ion diffusion, facilitating the movement of ions within the electrolyte^29,30^. However, this distinguishing feature is mostly dependent on and varies with the type and grade of paper used. The Whatman #1 filter paper is the most used substrate in paper-based fuel cells with all the required features, such as excellent porosity, pore size, and water uptake rate, etc. However, its capillary action is limited by electrolyte concentration. When loaded with a higher electrolyte concentration, its capillary action fails. To overcome this, the glass fiber (GF) filter paper was used. These are made from fine glass fibers and offer several advantages such as high concentration loading, high temperature resistance, improved absorbent properties, durability, and higher flow rates^31^. The utilization of fine glass fibers as a substrate within the architecture of a paper-based fuel cell has the potential to augment capillary action and hence, overall performance, particularly in scenarios involving elevated electrolyte concentrations. Nevertheless, it is crucial to conduct experiments and refine the dimensions, and organization of the fibers to attain the intended levels of efficiency and durability in fuel cells. Furthermore, it is imperative to establish appropriate bonding or integration of the glass fibers within the fuel cell structure to guarantee stability and optimize the utilization of capillary action.To further investigate the comparative liquid transport properties of GF and standard Whatman #1 paper, supplementary tests were conducted by introducing a paper strip measuring 10 cm × 0.7 cm into the microfluidic paper channel. Additional experiments were performed by immersing one end of a paper strip into a dye solution, and a brief video recording (SI-V1) and time-lapse images were shown in (Fig. 3a–g) captured for both papers to explore the phenomenon of capillary action. The wicking performance of different paper substrates was evaluated over a fixed distance of 5 cm to compare their fluid transport characteristics. As observed in Fig. 3f, Whatman #1 filter paper exhibited slower capillary action, taking approximately 15 min to wick the fluid across the entire length. In contrast, the Glass Fiber (GF) substrate in Fig. 3g, demonstrated significantly faster wicking, covering the same distance in just 2.2 min, highlighting its superior capillary flow properties. These results reveal that the GF paper has a faster flow rate than Whatman #1 paper, which improves electrolyte replenishment at the cathode catalyst layer and reduces local mass-transport limitations during operation. Although the dissolved oxygen concentration was not directly measured in the present work, the faster capillary-driven electrolyte transport enabled by the GF substrate is expected to promote improved reactant access and reduce concentration polarization at the cathode during operation. This continuous electrolyte supply is expected to improve ionic transport and facilitate electrochemical redox reactions, making GF a more effective substrate for microfluidic Al–air fuel cell operation^1^.Although the nominal pore size of Whatman #1 (11 μm) is larger than that of GF/A (1.6 μm), electrolyte transport in fibrous substrates is governed not only by pore diameter but also by pore interconnectivity, void fraction, tortuosity, wettability, and structural stability under alkaline conditions. Therefore, the markedly faster wicking observed for GF (2.2 min over 5 cm, compared with 15 min for Whatman #1) indicates a higher effective permeability and more favourable electrolyte transport network. The improved performance of GF is attributed to its superior capillary-driven transport and alkaline durability, as supported by the wicking experiment and electrochemical results, rather than by direct porosity quantification. Quantitative characterization of substrate porosity or permeability was not performed; therefore, the transport behaviour is interpreted based on the observed wicking characteristics and the corresponding electrochemical performance.

**Fig. 4:**
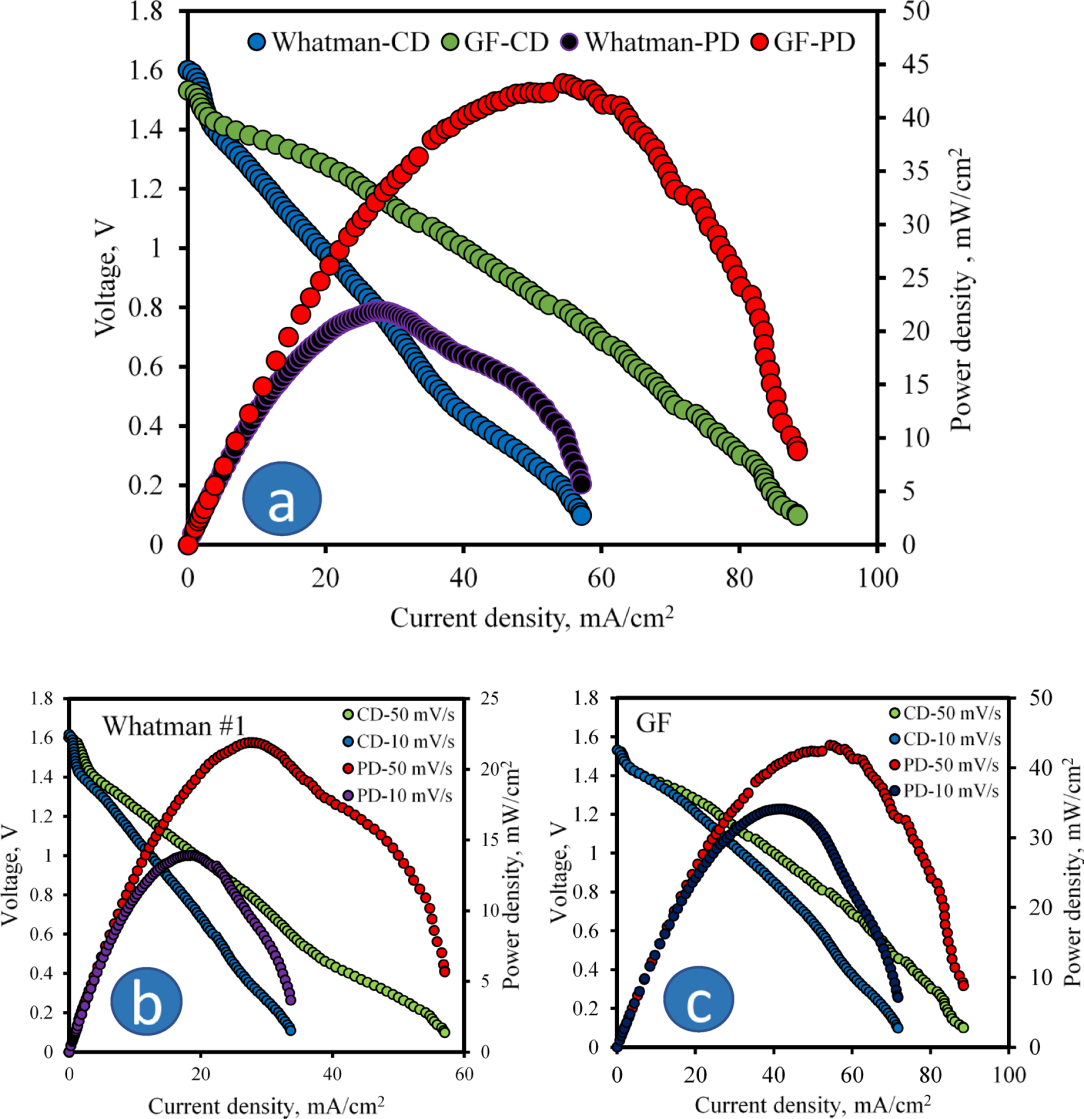
(**a**) Comparative polarization and power density curves of µA–aFC using Whatman #1 and glass fiber (GF) substrates using 2 M KOH electrolyte. (**b**) Polarization and power density curves of Whatman #1 substrate at different scan rates (50 and 10 mV/s). (**c**) Polarization and power density curves of GF substrate at different scan rates (50 and 10 mV/s) under identical conditions, showing comparatively improved performance retention at lower scan rate.

The comparative polarization analysis was carried out with Whatman #1 and GF paper with 2 M KOH electrolyte. As shown in Fig. 4a, the GF paper µA-aFC achieved peak power density (PPD) of 43.87 mW/cm^2^, which is nearly double that obtained with the conventional Whatman #1 substrate. Hence, GF paper was chosen as a substrate for the subsequent analysis. In addition, the maximum current density (MCD) for GF paper was found to be 90 mA/cm^2^ compared with of 55 mA/cm^2^, displaying the superior capillary properties of GF paper. The higher current density is due to a faster flow rate in GF paper, enables more efficient ion transport toward the electrode surface and consequently accelerates electrochemical reaction kinetics. The higher PPD further indicates improved electrode–electrolyte interaction when the GF substrate is used. In addition to improved electrolyte transport, enhanced wetting of the catalyst layer by the electrolyte within the porous GF substrate may also contribute to the observed increase in electrochemical activity and power density. However, catalyst wetting was not directly quantified in the present study, and its contribution is therefore discussed qualitatively. From a fabrication perspective, the laser-engraved GF substrate is also compatible with scalable manufacturing approaches. Laser patterning enables rapid and precise formation of microfluidic features without the need for masks or complex lithographic processing. In addition, commercially available GF sheets can be processed in batch formats, suggesting the potential of this substrate architecture for larger-scale production of paper-based Al–air fuel cells.

To further evaluate the influence of scan rate and to approach quasi steady-state conditions, polarization measurements were conducted at 10 mV/s and compared with those obtained at 50 mV/s for both Whatman #1 and GF substrates using 2.5 M KOH electrolyte (Fig. 4b and c). This intermediate electrolyte concentration was selected to more clearly capture transport-limited behaviour under varying scan rate conditions. A reduction in peak power density and current output is observed at the lower scan rate for both systems, which can be attributed to increased mass transport limitations under near steady-state conditions. Notably, the GF-based cell exhibits comparatively smaller performance degradation and sustains higher current density than the Whatman-based system. This trend suggests improved electrolyte transport and reduced transport limitations in the GF substrate, consistent with its enhanced capillary-driven transport characteristics.

### Concentration study

 In paper-based fuel cells, the electrolyte concentration has great significance as it affects the performance and charge transfer efficiency of the fuel cell. The performance of fuel cell depends on the pH (acidic/alkaline/neutral), nature (liquid or solid), and concentration of the electrolyte^32^. In Al-air fuel cells/batteries, alkaline electrolytes, such as potassium hydroxide (KOH) and sodium hydroxide (NaOH), are the two most prominently used electrolytes^33^. However, KOH is mostly preferred due to its properties such as higher ionic conductivity, higher oxygen diffusion coefficient, faster kinetic reaction, and inferior viscosity^34,35^. These value-added properties contribute towards improving the overall cell performance. Hence, KOH and NaOH were used as electrolytes, with concentrations ranging from 0.5 M to 5 M for each electrolyte to optimize the ideal concentration for the proposed cell. The polarization measurements were performed by scanning the cell voltage from the open-circuit potential (OCP) down to 0.1 V, where 0.1 V represents the lowest applied voltage to avoid instability or possible cell collapse near short-circuit conditions. Therefore, the maximum current density (MCD) corresponds to the current measured at 0.1 V, while the peak power density (PPD) was determined from the maximum value of the corresponding power density curve within the tested voltage range for different electrolyte concentrations of KOH and NaOH.

**Fig. 5:**
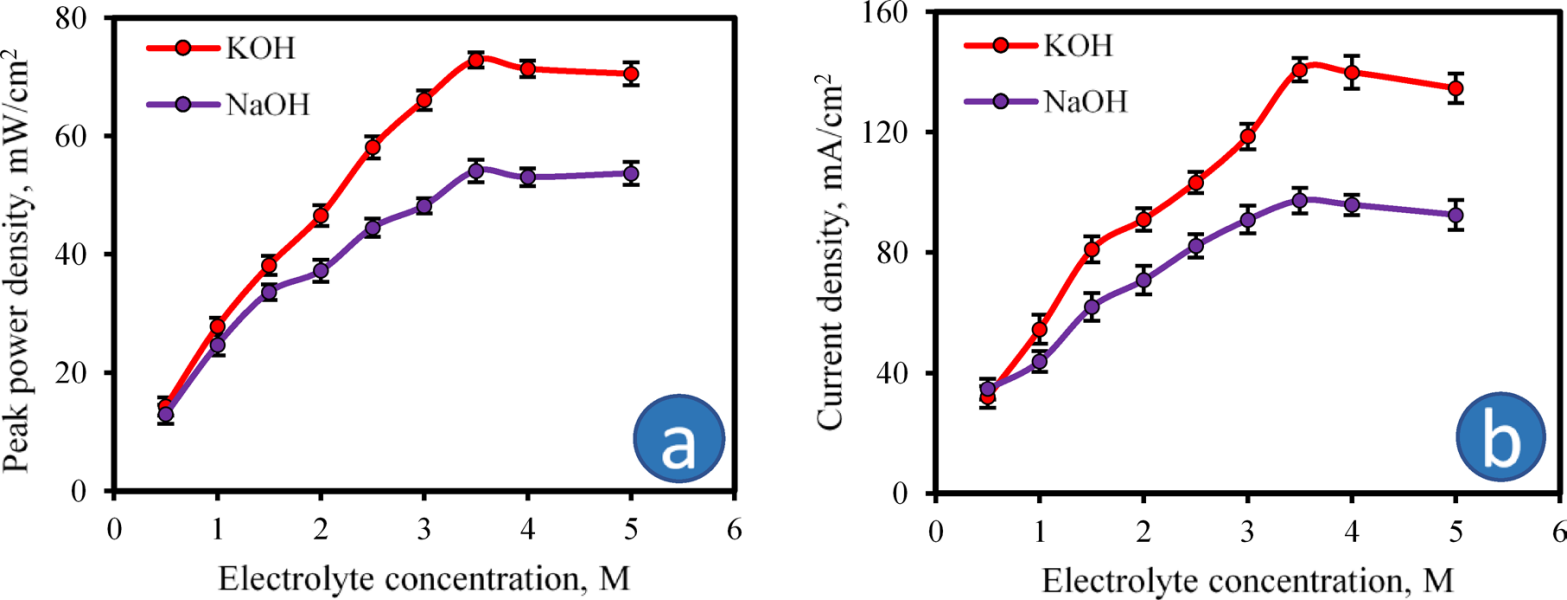
Performance investigation of GF paper-based µA-aFC with different electrolytes concentrations (**a**) Peak power density (PPD) measurement using KOH and NaOH, (**b**) Maximum current density (MCD) measurement at 0.1 V using KOH and NaOH electrolytes.

As shown in Fig. 5, KOH and NaOH display similar trends at different concentrations. The PPD and MCD increase drastically from 0.5 to 3.5 M, and gradually decrease on further increasing the concentration to 5 M. Several factors affect the stabilization or decrease in peak power density at higher electrolyte concentrations in fuel cells. Initially, increasing electrolyte concentration enhances ionic conductivity, leading to higher reaction rates at electrode surfaces and increased power density. However, beyond a certain threshold, further increases in concentration provide decreasing returns in ionic conductivity improvement. At higher electrolyte concentrations, increased ionic strength and electrolyte viscosity can influence ion transport near the electrode surface and may lead to mass transport limitations, which restrict reactant and product diffusion and thus limit further improvement in power density. Furthermore, electrode surfaces may become ionically saturated, limiting the number of active reaction sites. In addition, excessive electrolyte concentration may alter water distribution and ion mobility within the porous electrode structure, which can adversely affect electrochemical kinetics and overall cell performance^36,37,38^. Achieving optimal performance therefore requires a balance between electrolyte conductivity and mass transport conditions, suggesting that factors beyond electrolyte concentration also influence the overall fuel cell performance^39^.Overall, the cell displayed better activity with KOH as an electrolyte, as compared to NaOH (Fig. 5a and b). This behaviour corresponds to increasing viscosity with an increase in electrolyte concentration, which reduces electrolyte conductivity. The pores of the filter paper become clogged with the higher number of ions slowing down the transportation of the electrolyte. The internal resistance also increases simultaneously and plays a significant factor leading to a decrease in ion diffusion velocity in fuel cells.

### Electrochemical impedance spectroscopy (EIS) study

**Fig. 6:**
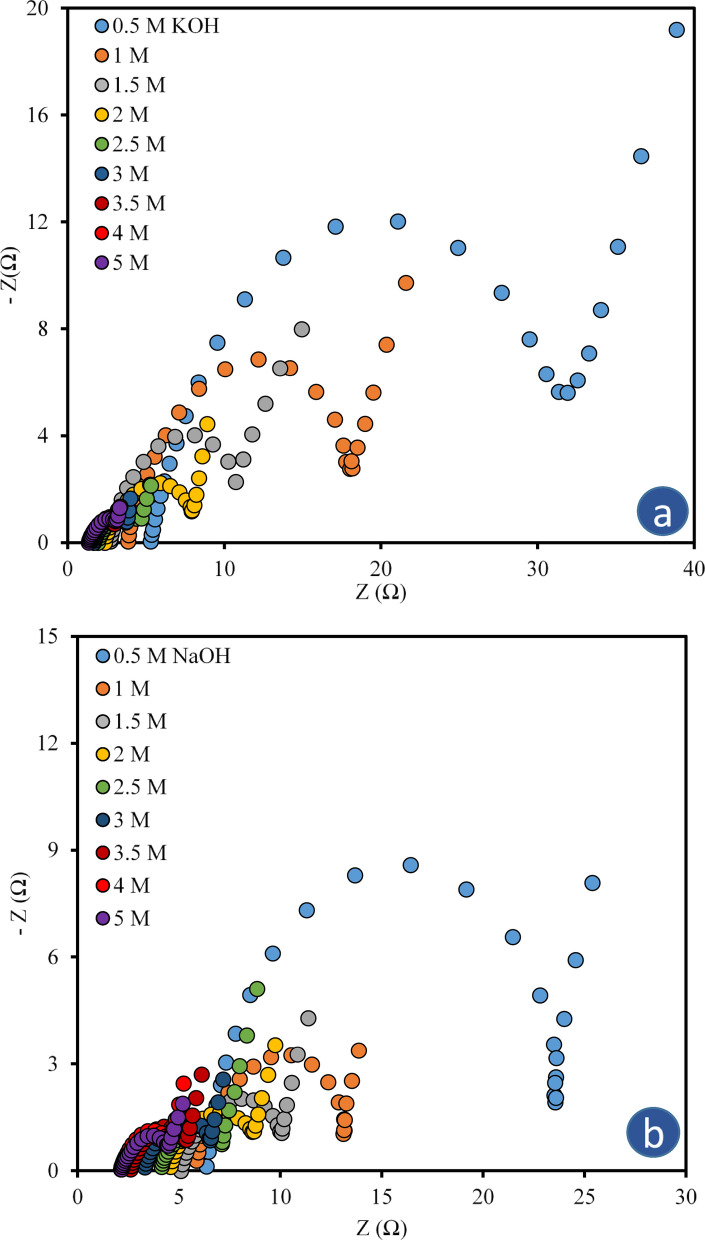
EIS analysis of GF paper-based µA-aFC with different electrolytes concentrations (**a**) KOH, (**b**) NaOH.

The EIS study was performed at a broad frequency spectrum with zero DC current to understand the electron transfer kinetics and the sources of performance loss mechanisms in the Al-air Fuel cell^40^. The EIS analysis enabled estimation of the cell charge transfer resistance (R_ct_), electrolyte resistance (Rs), linear Warburg impedance, double-layer capacitance (C_dl_), and other ohmic losses that contribute to the overall internal resistance of a fuel cell^41^. The Nyquist plot of the cell (Fig. 6) demonstrates how these values vary depending on the electrolyte employed, such as KOH and NaOH, and their concentration. The fuel cell electrolyte resistance (R_s_) significantly decreased from 5.45 Ω to 1.32 Ω while increasing the concentration of KOH from 0.5 M to 3.5 M, then slightly reduced to 1.295 Ω and 1.3 Ω for 4 M and 5 M KOH respectively, as shown in Fig. 6a. This trend in Rs is consistent with that observed for MCD and PPD (Fig. 5). The value of Rs shows similar trend with NaOH electrolyte also, i.e. it reduced from 6.53 Ω for 0.5 M to 2.71 Ω for 3.5 M, then decreased slightly to 2.1 Ω and 2.0 Ω for 4 M and 5 M NaOH (Fig. 6b).

Although the porosity of the substrates was not directly quantified in the present study, the lower electrolyte resistance (Rs) observed for the GF substrate is consistent with improved electrolyte transport through its interconnected porous network. In porous electrochemical systems, the effective ionic conductivity of the electrolyte within the porous medium is governed by structural parameters such as porosity and tortuosity, which are commonly described using Bruggeman-type relations^42,43^. Consequently, substrates with more open and interconnected pore structures can facilitate enhanced ionic transport and reduced ohmic resistance. The observed reduction in Rs is therefore qualitatively attributed to improved capillary-driven electrolyte transport and ionic conduction within the GF substrate.

This behaviour indicates that the effects of reduced R_s_ and increased hydroxyl $\:(OH^{ - } )$ ions contribute collectively to the enhancement of cell performance when the KOH/NaOH concentration was increased from 0.5 to 3.5 M. However, due to increased electrolyte viscosity and low diffusivity of hydroxyl ions, no additional gains in cell performance could be found at higher concentrations of 4 and 5 M. Although Rs remains relatively low at higher electrolyte concentrations, the overall cell performance does not continue to increase because concentrated alkaline electrolytes can hinder oxygen transport and cathodic reaction kinetics. Increased viscosity and reduced oxygen diffusivity introduce mass-transport limitations at the cathode, which can offset the benefit of low ohmic resistance and lead to saturation in power density beyond 3.5 M^44,45^. In addition, oxygen transport at the air cathode can also contribute to mass-transport limitations, particularly at higher electrolyte concentrations where increased viscosity and reduced oxygen diffusivity may restrict oxygen access to the catalyst layer^46^.

Table 2 summarizes detailed comparative EIS data for both KOH and NaOH with various concentrations. To investigate the diffusion and mass transport-limited behaviours of cells at different concentrations, the diffusion-based impedance Rd has been calculated from the linear Warburg impedance and double-layer capacitance (Cdl) using Zfit curve. As shown in Table 2, Rd decreases with increasing electrolyte concentration. This trend is generally associated with improved ion mobility, which allows ions to diffuse more readily through the electrolyt^25,47^. A corresponding increase in double-layer capacitance (Cdl) was also observed. This increase in Cdl may be associated with changes in ion distribution and interfacial charge storage behaviour at higher electrolyte concentrations. However, the thickness of the electrical double layer was not directly measured, and the observed trend is interpreted qualitatively based on the EIS response^48^.

**Table 2:** Electrolyte resistance (Rs), charge transfer resistance (Rct), diffusion-based impedance (Rd) and double-layer capacitance (Cdl) of GF paper-based µA- aFC with different electrolyte concentrations.

Concentration (M)	KOH				NaOH			
	Rs (Ω)	Rct (Ω)	Rd (Ω)	Cdl(µF)	Rs (Ω)	Rct (Ω)	Rd (Ω)	Cdl(µF)
0.5	5.45	15.23	124	2.9	6.53	16.41	154	3.3
1	4.2	13.92	109.8	6.8	6.01	6.6	113	7.5
1.5	3.5	7.2	72.23	14.9	5.09	6.6	76.81	15.59
2	2.5	2	64.8	18.23	4.5	3.7	69.43	20.98
2.5	2.1	2.4	56.34	25.76	4.23	2.65	48.43	23.15
3	1.7	2.2	42.77	33.67	3.39	3.2	43.35	34.78
3.5	1.32	1.9	39.89	40.55	2.71	2.71	45.4	46.59
4	1.295	2.2	41.67	51.86	2.1	2.56	47.01	54.81
5	1.3	1.91	40.11	59.12	2	2.4	45.31	63.05

### Polarization and stability analysis

It is important to carry out polarization studies for determining performance optimization and evaluating fuel cell performance as well as understanding more about the electrochemical properties, overpotentials and operating windows. Polarization studies in this sense were carried out using KOH/NaOH at concentrations of 0.5 M to 5 M. The study was carried out using I-V characterizations whereby the voltage was swept from open circuit potential (OCP) to 0.1 V at a rate of 50 mV/s and the resultant power density calculated by multiplying current and voltage. The KOH as well as NaOH concentrations were varied from 0.5 to 3.5 M. The highest power density and current density of 72.89 mW/cm^2^ and 143.76 mA/cm^2^ were obtained with a concentration 3.5 M KOH. The power density and current density remained constant upon further increasing the concentration to 4 M and 5 M. A similar trend for PPD and MCD was noticed for NaOH electrolyte and is illustrated comprehensively in Table 3. A brief video has been included in SI-V2 to demonstrate the potential of developed GF paper-based µA-aFC for real-time application by integrating laboratory-scale low-powered devices. However, detailed comprehensive polarization graphs for KOH and NaOH electrolytes at all concentrations are shown in Fig. S1.

**Fig. 7:**
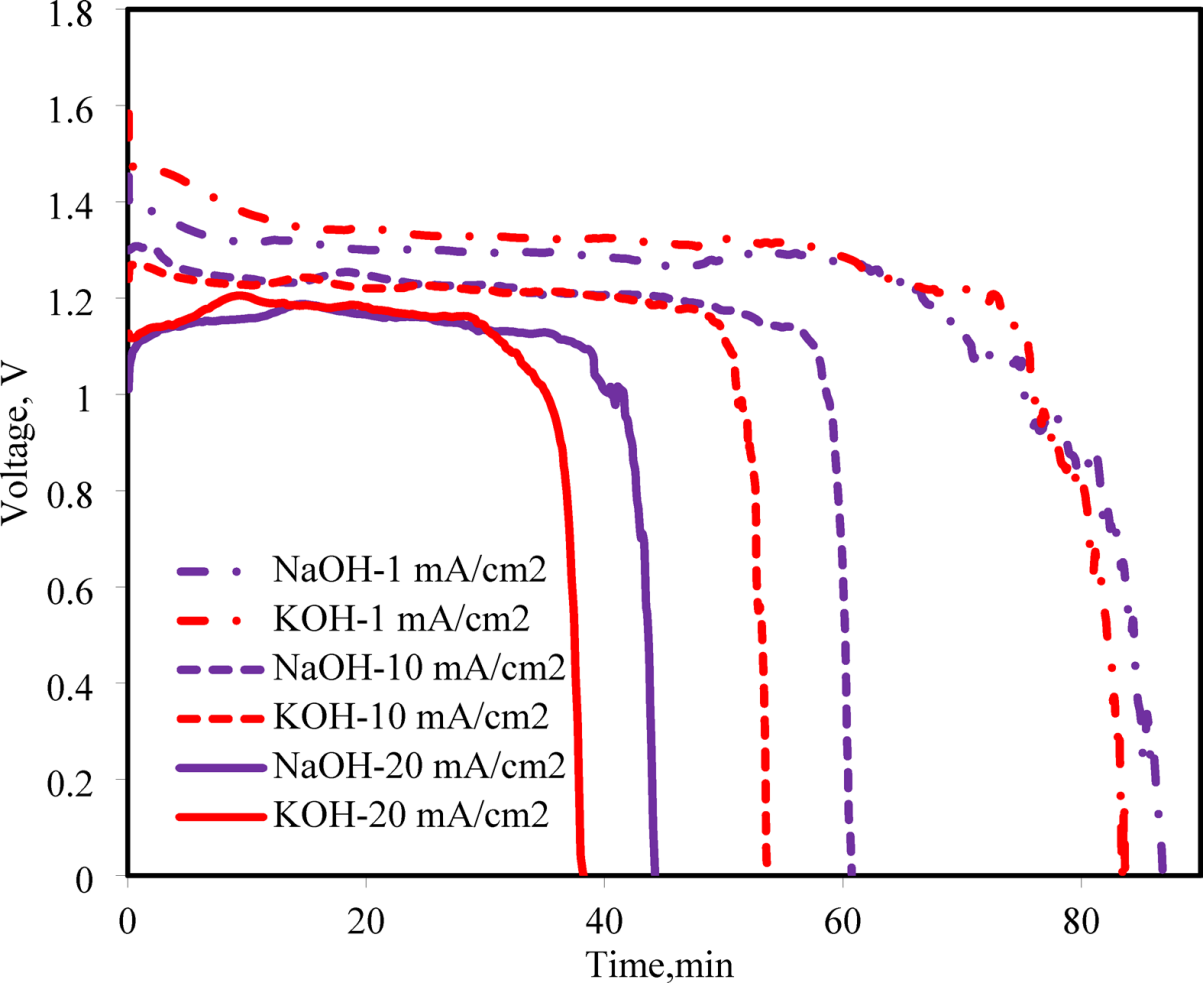
Comprehensive discharge studies with 3.5 M KOH/NaOH electrolytes at a constant 1, 10, and 20 mA/cm^2^ current density.

To study the stability of the developed cell with optimum 3.5 M (KOH/NaOH) concentrations, discharge studies were carried out at constant current densities of 1,10 and 20 mA/cm^2^. The discharge behaviours of the cell with respect to 3.5 M KOH and NaOH are shown in Fig. 7. The study carried out with 5.8 mg of Al as anode and 3.5 M KOH electrolyte, sustained for 84 min at a constant current density of 1 mA/cm^2^, 54 min at 10 mA/cm^2^, 38 min with 20 mA/cm^2^. Similarly sustained for 87 min at 1 mA/cm^2^, at 10 mA/cm^2^ for 60 min and at 20 mA/cm^2^ for 44 min with 3.5 M NaOH. From the analysis, it is observed that the cell using NaOH electrolyte operates for a longer duration than the KOH-based cell despite producing lower power output. This behaviour can be mainly attributed to the slower corrosion rate of aluminium in NaOH and differences in electrolyte viscosity and ionic conductivity^2^. However, the slight decrease in the cell voltage during initial phases was due to continuous consumption of anode (leading to the formation of Al and hydroxyl ion). Towards the end of the discharge curve sudden drop in voltage is observed, due to the complete consumption of the anode, and thus the reaction ceases to stop.

**Table 3:** Comprehensive polarization performance of GF paper based µA-aFC with different electrolytes (KOH/NaOH) concentration.

Conc. (M)	KOH			NaOH		
	OCP	MCD	PPD	OCP	MCD	PPD
0.5	1.65 ± 0.15	34.97 ± 0.78	14.26 ± 0.45	1.63 ± 0.11	30.15 ± 0.58	14.93 ± 0.52
1	1.52 ± 0.18	59.56 ± 0.68	29.46 ± 0.51	1.58 ± 0.19	43.86 ± 0.69	24.68 ± 0.45
1.5	1.60 ± 0.11	79.58 ± 0.82	38.06 ± 0.55	1.60 ± 0.15	61.95 ± 0. 78	33.58 ± 0.63
2	1.61 ± 0.25	90 ± 0.89	43.87 ± 0.63	1.63 ± 0.25	70.88 ± 0.99	37.2 ± 0.75
2.5	1.58 ± 0.22	103.33 ± 0.98	54.56 ± 0.75	1.58 ± 0.22	82.2 ± 1.08	44.48 ± 0.85
3	1.55 ± 0.17	118.55 ± 0.87	66.61 ± 0.67	1.55 ± 0.17	90.95 ± 1.01	48.19 ± 0.95
3.5	1.51 ± 0.12	143.76 ± 1.14	72.89 ± 0.66	1.51 ± 0.12	97.34 ± 1.23	54.11 ± 0.75
4	1.49 ± 0.11	139.86 ± 1.22	72.42 ± 0.77	1.49 ± 0.11	95.9 ± 1.15	53.05 ± 0.89
5	1.50 ± 0.14	134.56 ± 1.34	72.03 ± 0.72	1.50 ± 0.14	92.56 ± 1.20	53.69 ± 0.98

OCP: Open circuit potential, V; (MCD): Maximum current density, mA/cm^2^; PPD: Peak power density, mW/cm^2^

## Conclusions

This study demonstrates the successful enhancement of microfluidic aluminium–air fuel cell (µA–aFC) performance through the integration of glass fiber (GF) paper as a substrate for electrolyte transport. Compared to conventional Whatman #1 filter paper, GF paper exhibited superior capillary action, more open fibrous network, and structural and chemical durability in alkaline media, resulting in significantly improved ionic conductivity and redox kinetics. The µA–aFC employing GF paper and 3.5 M KOH achieved a peak power density of 72.89 ± 0.66 mW/cm² and a maximum current density of 143.76 ± 1.14 mA/cm² nearly double that of standard substrates. The study systematically investigated substrate behaviour, electrolyte concentration effects, and electrochemical impedance characteristics to identify optimal operating conditions. These findings validate GF paper as a high-performance alternative for sustainable, paper-based energy systems. By addressing limitations in electrolyte transport, this research fills a critical gap in µA–aFC development and opens avenues for future design of durable, scalable, and efficient fuel cells for portable and off-grid applications. Future research could leverage data-driven and machine learning optimization strategies to swiftly evaluate substrate-electrolyte pairings, thereby boosting power density and operational longevity. Additionally, coupling these µA–aFCs with energy-efficient IoT devices and wireless sensors would enable their evolution into autonomous, self-sustaining systems for decentralized environmental and health monitoring applications.

## Supplementary Information

Below is the link to the electronic supplementary material.

Supplementary Material 1

Supplementary Material 2

Supplementary Material 3

## Data Availability

Data is included within this manuscript.
