# Supplementary material for: Substrate-driven optimization of microfluidic aluminum–air fuel cells: a comparative study of glass fiber vs. cellulose paper
Source: Sci Rep. 2026 May 9;16:19784. doi: 10.1038/s41598-026-50431-3 (PMC13315794; doi:10.1038/s41598-026-50431-3)
Supplement: Supplementary file 3 — Supplementary Material 3 [file 41598_2026_50431_MOESM3_ESM.docx]

**Substrate-Driven Optimization of Microfluidic Aluminum–Air Fuel Cells: A Comparative Study of Glass Fiber vs. Cellulose Paper**

**^1*^Purshottam J. Assudani, ^2^R Lavanya, ^3^Srinivas Samala, ^3^Ch.Rajendra Prasad, ^4^Karthik M, ^5^Prakash Rewatkar, ^6^Manish Bhaiyya, and ^7*^Madhusudan B. Kulkarni**

^1^School of Computer Science and Engineering, Ramdeobaba University, Nagpur, Maharashtra, India.

^2^Department of Computing Technologies, SRM Institute of Science and Technology, Kattankulathur, Tamil Nadu, India.

^3^Department of ECE, SR University, Warangal-506371, Telangana, India.

^4^Department of Electrical and Electronics Engineering, Kongu Engineering College, Perundurai, Erode, Tamil Nadu, India.

^5^Department of Mechanical Engineering, Israel Institute of Technology, Haifa 3200003, Israel.

^6^Department of Electronics and Communication Engineering, Shri Sant Gajanan Maharaj College of Engineering, Shegaon 444203, Maharashtra, India.

^7^Manipal Institute of Technology, Manipal Academy of Higher Education (MAHE), Manipal, India.

***Corresponding author**: [assudanipj@rknec.edu](mailto:assudanipj@rknec.edu); [madhusudan.kulkarni@manipal.edu](mailto:madhusudan.kulkarni@manipal.edu)

|  |
| --- |
|  |
| Fig. S1 Detailed polarization plots for KOH and NaOH electrolytes at all concentrations |
